# All-cause and AIDS-related mortality among people with HIV across Europe from 2001 to 2020: impact of antiretroviral therapy, tuberculosis and regional differences in a multicentre cohort study

**DOI:** 10.1016/j.lanepe.2024.100989

**Published:** 2024-06-25

**Authors:** Christian Kraef, Erich Tusch, Sabine Singh, Lars Østergaard, Gerd Fätkenheuer, Antonella Castagna, Santiago Moreno, Katharina Kusejko, Bartosz Szetela, Anastasiia Kuznetsova, Janez Tomažič, Jovan Ranin, Robert Zangerle, Fredrik Mansson, Giulia Marchetti, Stéphane De Wit, Amanda Clarke, Jan Gerstoft, Daria Podlekareva, Lars Peters, Joanne Reekie, Ole Kirk, the EuroSIDA Study Group

**Affiliations:** a Department of Infectious Diseases, Rigshospitalet, Denmark; b CHIP, Centre of Excellence for Health, Immunity and Infections, Rigshospitalet, Copenhagen, Denmark; c Aarhus University Hospital, Skejby, Denmark; d Department I of Internal Medicine, University Hospital of Cologne, Cologne, Germany; e Infectious Diseases, San Raffaele Scientific Institute, Milano, Italy; f Department of Infectious Diseases, Hospital Universitario Ramón Y Cajal, Madrid, Spain; g Division of Infectious Diseases, University Hospital Zurich, University of Zurich, Zurich, Switzerland; h Wroclaw Medical University, Wroclaw, Poland; i Kharkov State Medical University, Kharkov, Ukraine; j Clinic for Infectious Diseases, Ljubljana University Medical Center, Ljubljana, Slovenia; k Infectious and Tropical Diseases University Hospital, Clinical Centre of Serbia, Belgrade, Serbia; l Medical University of Innsbruck, Innsbruck, Austria; m Skåne University Hospital, Clinical Infection Medicine, Department of Translational Medicine, Lund University, Malmö, Sweden; n Dept of Health Sciences, Clinic of Infectious Diseases, ASST Santi Paolo e Carlo, Italy; o CHU Saint-Pierre, Université Libre de Bruxelles, Brussels, Belgium; p Royal Sussex County Hospital, and Brighton & Sussex Medical School, Brighton, UK; q Department of Respiratory Medicine and Infectious Diseases, Copenhagen University Hospital, Bispebjerg, Denmark

**Keywords:** HIV, AIDS, Europe, Eastern Europe, Mortality, Tuberculosis, ART, Healthcare

## Abstract

**Background:**

All-cause and AIDS-mortality in Europe has been decreasing between 1996 and 2020. However, regional differences as well as their drivers remain unclear. This study investigates mortality differences and their drivers, including usage of and response to antiretroviral therapy (ART) and active tuberculosis (TB), among people with HIV across Europe.

**Methods:**

People with HIV enrolled in EuroSIDA were followed from 2001 through 2020. Immunologic-virologic status (IVS) was categorized as poor (CD4-cell count ≤350 cells/mm^3^ and viral load (VL) > 200 copies/ml), good (CD4 ≥ 500 and VL < 200), or intermediate (remaining combinations). Participants missing either CD4-cell count or VL were categorized as unknown. Regional differences in mortality were analyzed using multivariable Poisson regression with interaction analyses between regions of Europe and IVS, ART, or TB status.

**Findings:**

20,364 people with HIV were included: 13,715/20,346 (67.3%) from Western, 3020/20,364 (14.8%) from Central Eastern, and 3629/20,364 (17.8%) from Eastern Europe. At enrolment, median age was 40 years (inter-quartile range (IQR): 33–48), median CD4-cell count 449 cells/mm^3^ (IQR: 291–638), and most were male 14,993/20,346 (73.3%). A total of 2639 died during 192,591 person-years of follow-up (crude mortality rate 13.7/1000 person-years, 95% CI: 13.2–14.2), 519/2639 (19.7%) from AIDS (2.7/1000 person-years, 2.5–2.9). All-cause and AIDS-mortality rates decreased over time but remained higher in Eastern Europe after adjusting for confounders. Being off ART (aIRR 2.42; 95% CI 2.14–2.74), poor IVS (aIRR 4.2; 95% CI 3.39–5.20) and prior TB (aIRR 3.33; 95% CI 2.75–4.03) were associated with higher all-cause mortality. For all-cause mortality the effect of ART (test for interaction: p < 0.001) and IVS (p = 0.02), but not TB (p = 0.5) varied across regions.

**Interpretation:**

Overall mortality and AIDS-mortality rates decreased over time, but remained higher in Eastern Europe. A poor IVS, being off ART and prior active TB were related to higher mortality. Eastern Europe had the highest proportion of people with poor or unknown IVS, emphasizing the continued need to improve HIV care with a focus on early diagnosis, ART initiation, and adherence.

**Funding:**

EuroSIDA has received funding from ViiV Healthcare LLC, 10.13039/100017183Janssen Scientific Affairs, 10.13039/100005205Janssen R&D, Bristol-Myers Squibb Company, Merck Sharp & Dohme Corp, Gilead Sciences and the European Union’s Seventh Framework Programme for research, technological development and demonstration under EuroCoord grant agreement n˚ 260694. The study is also supported by a grant from the 10.13039/501100001732Danish National Research Foundation and by the International Cohort Consortium of Infectious Disease (RESPOND).

**Research in context**

**Evidence before this study**

We searched PubMed for articles published before May 1st, 2024 that investigated mortality and causes of death among people with HIV across regions in Europe. Our searches used the terms “HIV”, “HIV epidemic”, “Europe”, “mortality”, “cause of death”, “antiretroviral therapy”, and “tuberculosis” in different combinations. We included only studies published in English.

Previous research by the EuroSIDA and D:A:D cohort collaboration highlighted a decrease in all-cause and AIDS-related mortality across Europe in the time period up to the year 2011. Concurrently, the pattern of causes of mortality changed over time. People with HIV in Eastern Europe had almost triple the rate of AIDS-related deaths compared to those in Southern Europe, while Northern Europe saw higher rates of non-AIDS mortality, in particular related to malignancy. CD4-cell count, HIV-RNA viral load, social vulnerability, injecting drug use, and tuberculosis are important predictors of mortality across Europe. A recent cohort study of people living with HIV starting ART showed that despite decreasing all-cause and AIDS-mortality in Europe and Northern America between 1996 and 2020 these reductions were not seen in all sub-groups (e.g. women with injecting drug use).

**Added value of this study**

Our study, based on the large multicenter EuroSIDA cohort collaboration, is the first to demonstrate that overall and AIDS-related mortality among people with HIV has continued to decrease across all regions in Europe in the time period 2011–2020. Age-standardized mortality in Central Eastern Europe is now similar to Western Europe, while mortality rates remain higher in Eastern Europe. Poor immunological-virological status (IVS), being off antiretroviral therapy and tuberculosis continue to be drivers of mortality in all regions of Europe, the former two albeit to different degrees by region. Furthermore, the study provides information on person-time distribution of IVS across time-periods, stratified by region, showing that Eastern Europe continues to have the highest proportion of unknown or poor IVS, which may translate into continued higher mortality in the coming years. Although Eastern Europe is still behind, significant progress has been made in regards of IVS status.

**Implications of all the available evidence**

Our findings emphasize the need to further improve HIV care with a focus on early HIV diagnosis, early ART, ART adherence and retention in care, especially in Eastern Europe.

## Introduction

Over the past 30 years, significant global progress in HIV diagnosis, treatment, and prevention has been achieved, but recent years have seen a slowdown and increasing regional disparities in meeting the UNAIDS 95-95-95 targets, whereby 95% of people with HIV should be diagnosed, 95% of those diagnosed with HIV should be receiving antiretroviral therapy (ART), and 95% of all those receiving ART should achieve viral suppression.^1^ In Europe, ART uptake and viral suppression rates have improved steadily over the last two decades, but there is a notable variation between European regions. The cascade of care for people with HIV in Eastern Europe has been improving but still lags behind, with the lowest percentages of people with HIV on ART and achieving viral suppression.^2^^,^^3^ Previous research by the EuroSIDA collaboration highlighted a decrease in all-cause and AIDS-related mortality across Europe in the time period 2001–2010, but also underscored substantial regional differences in mortality rates and causes of death.^4^^,^^5^ This EuroSIDA study found that people with HIV in Eastern Europe had almost triple the rate of AIDS-related deaths compared to those in Southern Europe, while Northern Europe saw higher rates of non-AIDS mortality.^4^ In the Data Collection on Adverse events of Anti-HIV Drugs cohort (D:A:D), a prospective multi-cohort study of people with HIV under active follow up in 33 countries in Europe, USA and Australia, the proportion of AIDS-related deaths had decreased from 34% to 22% between 1999 and 2011, while the non-AIDS related cancers became the leading cause of death among people with HIV, rising from 9% to 23%.^6^ This shift was attributed to higher CD4-cell counts due to effective ART,^6^ highlighting that CD4 count and viral load are important predictors of health outcomes in people with HIV.^7^

Recent studies showed decreasing all-cause and AIDS-mortality in Europe and Northern America between 1996 and 2020. However, these reductions were not seen in all sub-groups (e.g. women with injecting drug use) and regional mortality differences as well as their drivers remain unclear.^8^^,^^9^ Social vulnerability plays a key role as a predictor of lower ART coverage and viral suppression as well as higher mortality for those on ART.^10^ In Eastern Europe and Central Asia social vulnerability is closely associated with injecting drug use, which concurrently remains a major mortality risk for people with HIV.^11^ Additionally, some areas in Eastern Europe remain hotspots for tuberculosis (TB)/HIV co-infections, historically driven by incarceration and additionally increasing mortality risk.^7^^,^^12^ In contrast the HIV-epidemic in Western Europe started earlier and has been found predominantly among men who have sex with men (MSM) and migrants.^13^ In previous studies people with HIV with TB in Eastern Europe faced a mortality risk up to four times higher than those in Western Europe, with a ten-year post-diagnosis mortality as high as 60%.^7^^,^^12^ Also, standards and integration of assessment and healthcare for TB and Non-communicable diseases have been found to be less systematically developed in Eastern Europe compared to Central Eastern and Western Europe.^14^^,^^15^

Therefore, the objective of this study was to compare mortality and causes of death among people with HIV across regions of Europe, and according to usage of and response to ART as well as to the presence of an active TB diagnosis.

## Methods

### Study design and participants

Participants were from the prospective observational EuroSIDA study.^13^ The study includes more than 24,500 participants with HIV-1 followed in 118 hospitals in 39 European countries and Israel. People with HIV aged 18 year or older were enrolled into 11 cohorts from 1994 onwards. Clinical and laboratory data were collected prospectively at clinical sites and submitted to the coordinating centre at least at yearly intervals. Detailed information about the EuroSIDA cohort has been published previously and is available online (www.chip.dk/Research/Studies/EuroSIDA).^13^

In the present study, baseline was defined as either 1st January 2001, or enrolment into EuroSIDA, which ever occurred later. Participants with gender missing or transgender were excluded due to very low numbers, as were participants without any follow-up data. Individuals were followed from baseline until the earliest of date of death, loss to follow-up (defined as two years after last clinical visit or CD4/viral load measurement), or December 31st, 2020.

### Outcome definitions

The primary outcome was death, with secondary focus on AIDS-related mortality. Death was ascertained by the participating clinics through information from sources such as hospital files, outpatient clinical chart, autopsy report, registry, obituary, patient's relatives or partner, patient's medical provider and nursing homes. AIDS-related deaths were identified using the Coding Causes of Death in HIV (CoDe) methodology or an algorithm previously developed for classification of unknown or missing causes of death.^16^^,^^17^ Deaths due to AIDS identified by either method were grouped together in analysis of AIDS-related mortality.

### Variable selection

The following variables were assessed: age, gender, geographic region, risk factors for HIV acquisition, time since HIV diagnosis, CD4-cell count, HIV RNA viral load (VL), ART status, Hepatitis C virus (HCV) status, history of active TB (i.e., TB disease, not latent TB/TB infection), history of AIDS-defining event, cardiovascular disease (CVD), diabetes, end-stage renal disease (ESRD), end-stage liver disease (ESLD), Non-AIDS defining malignancies (NADM) and smoking status.^18^ Baseline CD4-cell count and VL were defined as the measurement closest to baseline date within one year prior to baseline or, if unavailable, within three months post baseline. HCV positive was defined as either antibody-positive, RNA-positive, genotype test results, or HCV-active medication pre-baseline. The geographic regions were defined as West (Austria, Belgium, Denmark, Finland, France, Germany, Greece, Iceland, Ireland, Israel, Italy, Luxembourg, the Netherlands, Norway, Portugal, Spain, Sweden, Switzerland, and the UK), Central East (Bosnia-Herzegovina, Croatia, Czechia, Hungary, North Macedonia, Poland, Romania, Serbia and Slovenia), and East (Belarus, Estonia, Georgia, Latvia, Lithuania, Russia and Ukraine) Europe.^12^ People that had more than one potential mode of HIV acquisition (i.e., injecting drugs use (IDU)/homosexual, IDU/heterosexual) were grouped in the IDU risk category. The definitions of AIDS-defining events can be found on the EuroSIDA website (https://chip.dk/Research/Studies/EuroSIDA) and reflect the definitions the Centers for Diseases Control's list of AIDS-defining conditions.^19^ CVD, ESRD, ESLD and NADM are defined in the EuroSIDA study protocol, specifically the Standard Operating Procedure for data transfer (https://chip.dk/Portals/0/files/RESPOND/Study%20documents/RESPOND_EuroSIDA_SOP_Electronic_Version_7.0.pdf?ver=2023-07-18-150155-957&timestamp=1689685440820).

Time-varying covariates included: time-period under follow-up, age, immunologic-virologic status (IVS), defined as poor (CD4 ≤ 350 cells/mm^3^ & VL > 200 copies/mL), good (CD4 ≥ 500 & VL < 200), intermediate (remaining combinations), and unknown (missing either CD4 or VL information); history of active TB was classified as no history of TB, TB pre-baseline (or at baseline), and incident TB during follow-up; smoking status was collected via self-report and classified as never smoker, former smoker, current smoker, or unknown smoking history; ART status was defined as naïve, or experienced, currently on any treatment, or currently off treatment. Time-updating of time-varying co-variates was done monthly by using last observation carried forward, without interpolation of measurements.

### Statistical analysis

Baseline characteristics of all participants included in the analysis were summarized and presented for the entire cohort and stratified by European region. Differences in baseline characteristics between regions were estimated by ANOVA tests for continuous variables and chi square tests for categorical variables.

The crude incidence rates for all-cause mortality and AIDS-related mortality were estimated overall, and by the time periods 2001–2010, 2011–2015 and 2016–2020. The time period 2001–2010 was chosen for comparison of mortality (including AIDS-related mortality) as this period has been published previously.^17^ The follow-up time period 2011–2020 was then further stratified by five-year intervals due to changing European AIDS Clinical Society guidelines that since 2015 have recommended initiation of ART regardless of CD4-cell count.^20^ These are used by at least 80% of all clinics contributing data to EuroSIDA as the primary guideline according to a recent survey.^15^ Age standardized mortality rates were estimated using the age distribution of the entire follow-up period, with ages grouped into 10-year age groups, using Dobson's approach to calculate confidence intervals (Supplementary Table S3).^21^, ^22^, ^23^ Crude and age-standardized mortality rates were also stratified by region (Western, Central Eastern and Eastern Europe), as well as according to immunologic-virologic status (IVS) and TB status (all time-varying).

Poisson regression was used to assess the outcome death. All variables with a p < 0.1 in univariable analyses were included in the multivariable model. The variables considered included age, gender, geographic region, HIV transmission risk group, time-varying time period (2001–2010, 2011–2015 and 2016–2020), IVS, active TB, ART status, HCV status and smoking status. The multivariable Poisson-regression model for all-cause and AIDS-related mortality was adjusted for age, gender, mode of HIV transmission, region, and time-varying IVS, ART status, time-period under follow-up, active TB history, and smoking history (all p < 0.1).

In addition, we performed interaction analyses between region of Europe and each of ART-status, active TB history, and IVS. We also conducted interaction analyses between time period and active TB history. The interaction analyses were defined a-priori based on the co-variates of clinical and policy interest.

The extent of loss to follow-up was compared between Eastern, Central Eastern, and Western Europe to investigate differences in access to health care across the three regions. We estimated crude rates of loss to follow-up per region and time period.

### Sensitivity analysis

As there was the potential for the COVID-19 pandemic to bias our estimates, we conducted a sensitivity analysis using an earlier cut-off of 29th February 2020 to check the robustness of our results prior to the pandemic.

Analyses were performed using R version 4.2.0.^24^, ^25^, ^26^ Reported p-values are two sided.

### Ethical considerations

Informed consent and ethics committee approval were obtained in each participating center according to national guidelines. Full study details are available at https://chip.dk/Research/Studies/EuroSIDA.

### Role of the funding source

EuroSIDA has received funding from ViiV Healthcare LLC, Janssen Scientific Affairs, Janssen R&D, Bristol-Myers Squibb Company, Merck Sharp & Dohme Corp, Gilead Sciences and the European Union's Seventh Framework Programme for research, technological development and demonstration under EuroCoord grant agreement n˚ 260694. The participation of centres from Switzerland has been supported by The Swiss National Science Foundation (Grant 148522). The study is also supported by a grant [grant number DNRF126] from the Danish National Research Foundation and by the International Cohort Consortium of Infectious Disease (RESPOND). None of the funders had a role in writing this manuscript or the decision to submit for publication.

## Results

A total of 20,364 people with HIV were included in the study (after excluding 5 participants with insufficient gender data), with 192,591 person-years of follow-up (PYFU), corresponding to a median follow-up time of 8.1 years (IQR 4.4–15.0). Most (67.3%) were from Western Europe, followed by Eastern Europe (17.8%) and Central Eastern Europe (14.8%). Overall, there were 12.1% with advanced HIV-diseases (CD4 cell-count <200 cell/mm3). There were clear differences in baseline demographics and other patient characteristics across the regions (Table 1). In Eastern Europe, people with HIV were more likely to be female (42.1%), younger at baseline (median age 32 years), have had a shorter time with known HIV (3.2 years), and have acquired HIV through injection drug use (48.4%) or heterosexual contact (41.7%) compared to Western and Central Eastern Europe. They were more often ART naïve (41.8%) and had an unknown IVS (34.8%) at baseline, had a lower CD4 cell-counts (median 409 cells/mm^3^) and higher HIV-RNA viral loads (median 499 cp/ml). In total, 1.6% (n = 324) of all participants with HIV had incident active TB during follow-up, 0.7% (n = 93) in Western Europe, 1.6% (n = 47) in Central Eastern and 5.1% (n = 184) in Eastern Europe.

**Table 1:** Patient characteristics at baseline, overall and by region.

Characteristics	Overall (n = 20,364; 100%)	Western Europe (n = 13,715; 67.3%)	Central-Eastern Europe (n = 3020; 14.8%)	Eastern Europe (n = 3629; 17.8%)	p-value
Median baseline date		2004-01-16	2008-07-10	2008-12-11	
Median age (IQR)	40 (33–48)	42 (36–50)	35 (30–43)	32 (27–39)	<0.001
Years since HIV diagnosis	8.6 (3.9–14.9)	11.2 (6.4–16.5)	3.6 (1.5–6.5)	3.2 (1.3–6.1)	<0.001
Gender					
Male	14,993 (73.7%)	10,575 (77.1%)	2326 (77%)	2102 (57.9%)	<0.001
HIV transmission risk					
MSM	7529 (37%)	5967 (43.5%)	1334 (44.2%)	228 (6.3%)	<0.001
Injection Drug Use	5817 (28.6%)	3300 (24.1%)	774 (25.6%)	1755 (48.4%)	
Heterosexual Contact	5661 (27.8%)	3450 (25.2%)	698 (23.1%)	1514 (41.7%)	
Other/unknown	1344 (6.6%)	998 (7.3%)	214 (7.1%)	132 (3.6%)	
ART status					
On ART	16,584 (81.5%)	12,060 (87.9%)	2550 (84.4%)	1978 (54.5%)	<0.001
Off ART, not naïve	874 (4.3%)	672 (4.9%)	69 (2.3%)	133 (3.7%)	
ART naïve	2893 (14.2%)	983 (7.2%)	401 (13.3%)	1518 (41.8%)	
CD4-cell count (cells/mm^3^)					
Median	449 (291–638)	461 (301–657)	433 (280–620)	409 (259–588)	<0.001
CD4 < 200	2463 (12.1%)	1533 (11.2%)	396 (13.1%)	534 (14.7%)	<0.001
HIV RNA Viral Load (cp/mL)					
Median	49 (39–1718.8)	49 (31–890)	49 (39–751)	499 (74–21300)	<0.001
Immunologic-virologic status					
Poor (CD4 ≤ 350 & VL > 200)	2781 (13.7%)	1783 (13%)	383 (12.7%)	615 (16.9%)	<0.001
Intermediate	8988 (44.2%)	6307 (46%)	1409 (46.7%)	1273 (35.1%)	
Good (CD4 ≥ 500 & VL < 200)	5932 (29.1%)	4593 (33.5%)	860 (28.5%)	479 (13.2%)	
Unknown	2650 (13%)	1032 (7.5%)	368 (12.2%)	1262 (34.8%)	
AIDS history	5069 (24.9%)	3768 (27.5%)	662 (21.9%)	645 (17.8%)	<0.001
CVD history	403 (2%)	365 (2.7%)	29 (1%)	9 (0.2%)	<0.001
Diabetes history	682 (3.4%)	597 (4.4%)	71 (2.4%)	14 (0.4%)	<0.001
ESRD history	44 (0.2%)	39 (0.3%)	5 (0.2%)	0 (0%)	0.004
ESLD history	168 (1.2%)	168 (1.2%)	7 (0.2%)	22 (0.6%)	<0.001
NADM history	400 (2%)	362 (2.6%)	23 (0.8%)	15 (0.4%)	<0.001
Smoking history					
Never	4229 (20.8%)	2657 (19.4%)	881 (29.2%)	691 (19%)	<0.001
Former	8206 (40.3%)	5960 (43.5%)	1081 (35.8%)	1170 (32.2%)	
Current	3873 (19%)	1955 (14.3%)	722 (23.9%)	1204 (33.2%)	
Unknown	4043 (19.9%)	3143 (22.9%)	336 (11.1%)	564 (15.5%)	
TB history					
Never TB	19,247 (94.6%)	13,008 (94.9%)	2858 (94.7%)	3392 (93.5%)	<0.001
TB pre-baseline	1104 (5.4%)	707 (5.1%)	162 (5.3%)	237 (6.5%)	
Hepatitis C Virus (HCV)					
Ever HCV antibody positive at baseline	8105 (39.8%)	4875 (35.5%)	1040 (34.4%)	2200 (60.6%)	<0.001

IQR–Interquartile range; MSM–Men having sex with men; ART-Antiretroviral therapy; AIDS–acquired immunodeficiency syndrome; CVD–cardiovascular disease; ESRD-End stage renal disease; NADM–Non-AIDS defining malignancies; TB-tuberculosis; VL-HIV-RNA viral load, VL-HIV-RNA values that are below the lower limit of detection (LOD) are given a value of (LOD-1).

### Distribution of immunologic-virologic status across time

**Fig. 1:**
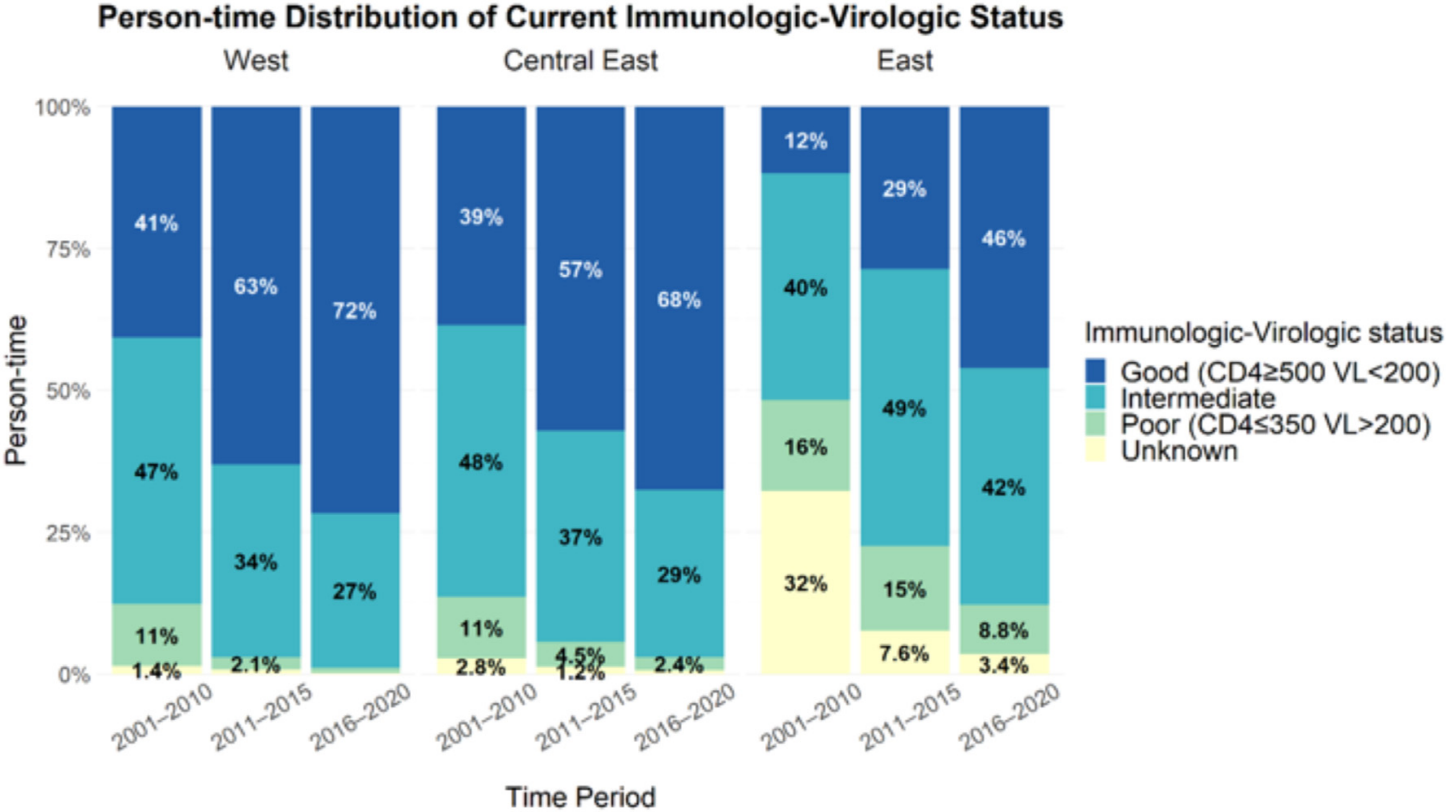
Person-time distribution of immunologic-virologic status across time-periods, stratified by region.

IVS improved over time across all regions (Fig. 1). The proportion of person-time with good IVS in the latest time period from 2016 to 2020 remained, however, much lower in Eastern Europe (46.1%, 3783/8202 PYFU), compared to Central Eastern (67.7%, 5946/8789 PYFU) and Western Europe (71.6%, 28531/39831 PYFU).

### Overall and AIDS-related mortality

Overall, 2639 people with HIV died during follow-up, corresponding to a crude mortality rate of 13.7/1000 PYFU (95% CI: 13.2–14.2). Of those, 519 (19.7%) died from AIDS-related causes (2.7/1000 PFYU, 95% CI 2.5–2.9%). AIDS-related causes were responsible for 14.6% (n = 271/1862) of deaths in Western Europe, 24.6% (n = 68/276) of deaths in Central Eastern and 35.9% (n = 180/501) of deaths in Eastern Europe.

**Fig. 2:**
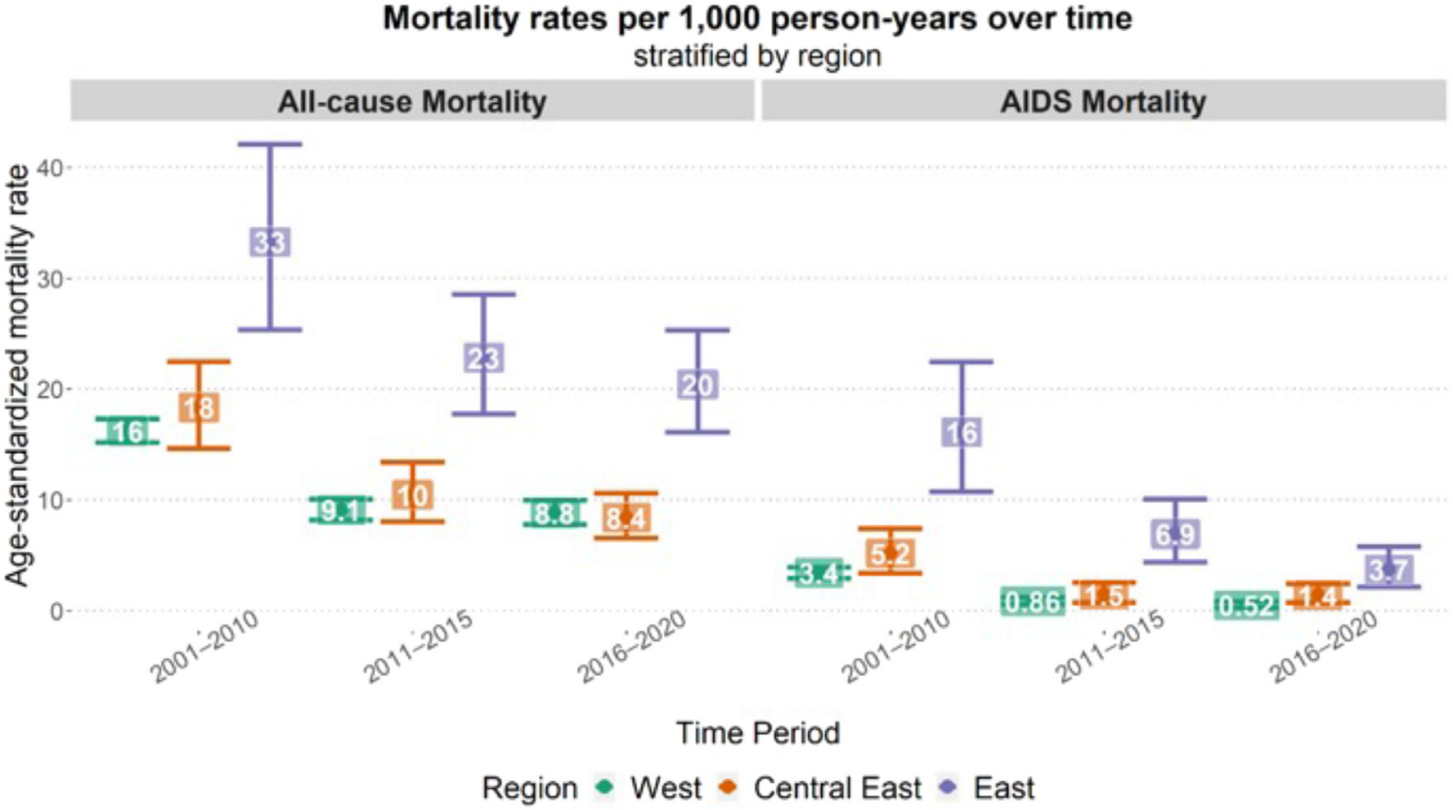
Age-standardized mortality rates (all-cause and AIDS mortality) per 1000 PYFU, stratified by region and time-period under follow-up.

In crude, age-standardized analyses all-cause and AIDS mortality declined over time across regions (Fig. 2). However, in these crude analyses both all-cause and AIDS-mortality remained highest in Eastern Europe in the most recent time period, whereas in this time period all-cause mortality rates in Central Eastern Europe appeared similar to those in Western Europe, and AIDS-mortality in Central Eastern Europe was only slightly higher than in Western Europe.

### Risk factors for all-cause mortality

Table 2 provides the results of the multivariable Poisson regression analysis for all-cause mortality. The most important risk factors for all-cause mortality in the adjusted multivariable model were poor IVS (vs good IVS; aIRR 7.61; 95% CI 6.68–8.68) or unknown IVS (aIRR 4.20; 95% CI 3.39–5.20), incident active TB during follow-up (aIRR 3.33; 95% CI 2.75–4.03), being off ART (aIRR 2.42; 95% CI 2.14–2.74), and receiving care in Eastern Europe (vs. Western Europe; aIRR 1.55; 1.40–1.71). Supplementary Table S2 shows adjusted risk factors for all-cause mortality stratified by region of care. sup.

**Table 2:** Risk factors for all-cause and AIDS-related mortality in univariable and multivariable adjusted Poisson regression models.

Risk Factor	All-cause		AIDS	
	IRR (95% CI)	aIRR (95% CI)	IRR (95% CI)	aIRR (95% CI)
Age				
Per 1 year older	1.03 (1.02, 1.03)	1.06 (1.06, 1.07)	0.98 (0.97, 0.99)	1.03 (1.02, 1.04)
Time Period (ref: 2001–2010)				
2011–2015	0.74 (0.68, 0.82)	0.79 (0.71, 0.87)	0.45 (0.36, 0.56)	0.54 (0.42, 0.68)
2016–2020	0.77 (0.7, 0.84)	0.77 (0.69, 0.85)	0.29 (0.22, 0.37)	0.45 (0.34, 0.6)
Gender (ref: Male)				
Female	0.71 (0.64, 0.78)	0.79 (0.71, 0.87)	0.75 (0.61, 0.93)	0.64 (0.5, 0.81)
HIV transmission risk (ref: MSM)				
IDU	2.19 (2, 2.39)	2.08 (1.87, 2.31)	2.3 (1.85, 2.85)	1.18 (0.91, 1.51)
Heterosexual contact	0.95 (0.85, 1.06)	1.01 (0.9, 1.14)	1.46 (1.16, 1.83)	1.2 (0.93, 1.55)
Other/unknown	1.2 (1.01, 1.42)	1.08 (0.91, 1.28)	1.17 (0.78, 1.76)	0.89 (0.59, 1.36)
Region (ref: Western)				
Central Eastern	0.79 (0.69, 0.89)	1 (0.88, 1.14)	1.33 (1.02, 1.74)	1.44 (1.09, 1.91)
Eastern	1.55 (1.4, 1.71)	1.5 (1.33, 1.69)	3.82 (3.16, 4.61)	2.25 (1.74, 2.9)
Immunologic/Virologic status (ref: Good)				
Poor	8.54 (7.63, 9.57)	7.61 (6.68, 8.68)	66.66 (45.08, 98.58)	37.63 (24.89, 56.87)
Intermediate	3.08 (2.79, 3.39)	2.82 (2.55, 3.12)	8.83 (5.94, 13.14)	6.82 (4.56, 10.19)
Unknown	4.26 (3.51, 5.18)	4.2 (3.39, 5.2)	44.06 (28.22, 68.79)	22.6 (14.04, 36.37)
Current ART history (ref: On ART)				
Off ART	3.91 (3.5, 4.37)	2.42 (2.14, 2.74)	7.38 (5.97, 9.13)	2.4 (1.91, 3.03)
ART naïve	1.5 (1.28, 1.76)	1.17 (0.98, 1.4)	3.52 (2.69, 4.62)	1.06 (0.78, 1.44)
Active TB history (ref: No history of TB)				
History of TB, pre-baseline	1.76 (1.53, 2.02)	1.45 (1.26, 1.67)	2.75 (2.08, 3.63)	2.27 (1.71, 3.03)
Incident TB during follow-up	5.55 (4.63, 6.65)	3.33 (2.75, 4.03)	23.27 (18.39, 29.45)	11.43 (8.75, 14.93)
Smoking history (ref: Never smoker)				
Former smoker	1.31 (1.15, 1.48)	1.15 (1, 1.31)	1.02 (0.77, 1.33)	1.02 (0.77, 1.36)
Current smoker	1.8 (1.6, 2.02)	1.55 (1.37, 1.76)	1.33 (1.05, 1.69)	1.07 (0.82, 1.39)
Unknown	0.84 (0.71, 0.98)	0.79 (0.67, 0.93)	0.74 (0.53, 1.03)	0.82 (0.58, 1.15)

IRR: univariate, unadjusted incidence Rate Ratio; aIRR: adjusted incidence Rate Ratio; 95% CI: 95% confidence interval; IDU: Injecting Drug Use; MSM: Men having sex with men; ART: Antiretroviral therapy; AIDS: acquired immunodeficiency syndrome; TB: tuberculosis; VL: viral load.

Multivariable models were adjusted for age, gender, mode of HIV transmission, region, and time-varying IVS, current ART, active TB, time-period under follow-up, and smoking status.

### Risk factor for all-cause mortality, stratified by time-period of follow-up

For all-cause mortality, there was a significant interaction between time-period and TB status (p < 0.001), indicating that the association between active TB and mortality changed over time.

The association between an active TB diagnosis during follow-up and mortality declined but was still significant (aIRR 2.26; 95% CI 1.53–3.23 in 2016–2020 compared to aIRR 2.50; 95% CI 1.74–3.59 in 2011–2015 and 5.27; 95% CI 4.01–6.93 between 2001 and 2010).

Furthermore, in the time-period 2011–2015, unknown IVS (vs. good IVS) was a strong predictor of mortality (aIRR 3.78; 95% CI 2.26–6.32). This association was not present in the most recent time period 2016–2020 (aIRR 1.93; 95% CI 0.86–4.33).

### Effect of ART-status, IVS and active TB on mortality across regions

**Fig. 3:**
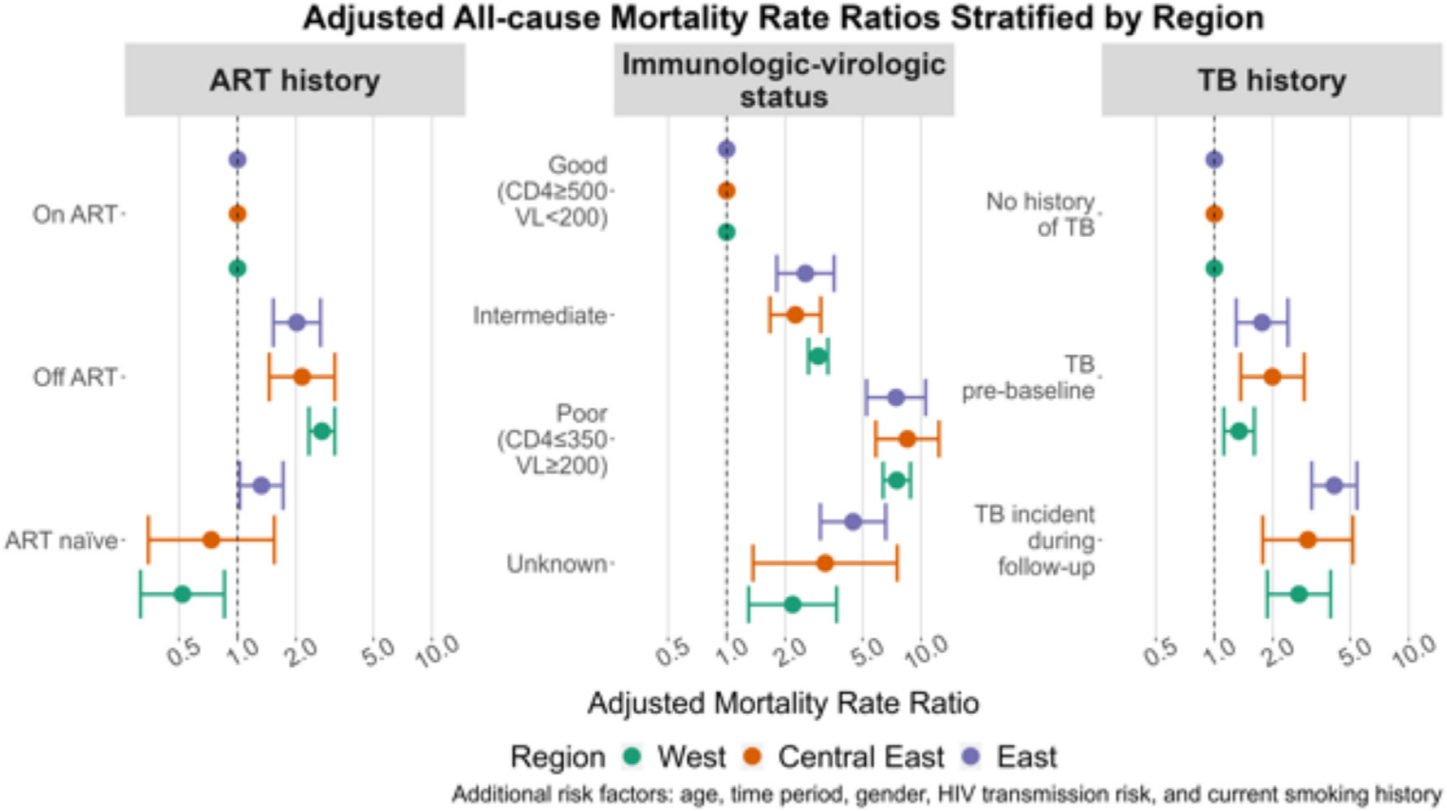
Adjusted all-cause mortality rate ratios for ART history, IVS and active TB history, stratified by region.

We also observed significant interactions on the risk of all-cause mortality between region of Europe and ART-status (p < 0.001 for interaction) and IVS (p = 0.001) but not for active TB (p = 0.22). Being off ART (vs. on ART) increased the all-cause mortality consistently in Eastern (aIRR 2.02; 95% CI 1.53–2.67), in Central Eastern (aIRR 2.15; 95% CI 1.46–3.16) and in Western Europe (aIRR 2.71; 95% CI 2.33–3.16) in stratified multivariable models (Fig. 3), whereas being ART-naïve (vs. on ART) was associated with a lower mortality in Western Europe (aIRR 0.52; 95% CI 0.32–0.86) and a higher mortality in Eastern Europe (aIRR 1.33; 95% CI 1.03–1.71).

Intermediate or poor IVS was associated with a substantially and consistently increased mortality across all regions. However, an unknown IVS status (vs. good IVS) was associated with a higher mortality in Eastern Europe (aIRR 4.47; 95% CI 3.03–6.58) compared to Western (aIRR 2.18; 95% CI 1.30–3.67) and Central Eastern Europe (aIRR 3.21; 95% CI 1.37–7.54).

### Risk factors for AIDS-related mortality

The most important risk factors for AIDS-related mortality in the adjusted multivariable model were being under HIV care in Eastern Europe (vs. Western, aIRR 2.25; 95% CI 1.74–2.90), poor IVS (vs. good IVS, aIRR 37.6; 95% CI 24.9–56.9), unknown IVS (vs. good IVS, aIRR 22.6; 95% CI 14.0–36.4), being off ART (vs. on ART, aIRR 2.40; 95% CI 1.91–3.03) and incident active TB after baseline (vs. no history of active TB, aIRR 11.4; 95% CI 8.75–14.9) (Table 2).

### Loss to follow-up by region

Overall loss-to follow-up was stable between 2001 and 2010 (IR 18.9/1000 PYFU; 95% CI 18–19.9) and 2011–2015 (IR 18.4/1000 PYFU; 95% CI 17.3–19.6) but increased in the most recent time-period, 2016–2020 (IR 34.8/1000 PYFU; 95% CI 33.3–36.4). Over the whole study period, the rate of loss to follow-up was highest in Eastern Europe (IR 34.7/1000 PYFU, 95% CI 32.4–37.2), compared to Western Europe (IR 22.2/1000 PYFU, 95% CI 21.4–23.0) and Central Eastern Europe (IR 19.9/1000 PYFU, 95% CI 18.2–21.7). These differences remained in the latest time-period from 2016 to 2020 (Eastern Europe: IR 49.3; 95% CI 44.6–54.4, Central Eastern Europe: IR 32.5; 95% CI 28.8–36.5 and Western Europe: IR 32.4; 95% CI 30.6–34.2).

### Sensitivity analysis

Sensitivity analysis using a cut-off date of February 29th 2020 to exclude the possibility of the COVID-19 pandemic impacting our results identified consistent risk factors of similar magnitude to the main analysis (Supplementary Table S4). However, we did observe a slightly larger decrease in risk of all-cause mortality for the time-period 2016–2020 compared to time-period 2001–2010 when the 10 months of the pandemic were excluded (aIRR 0.77 (95% CI 0.69, 0.85) in the main analysis to aIRR 0.65 (0.58, 0.73) in the sensitivity analysis).

## Discussion

In this unique cohort study including people with HIV across the whole European continent with nearly 200,000 person-years of follow-up, we show a reduction of all-cause and AIDS-mortality across all European regions over the last two decades. Age-standardized mortality in Central Eastern Europe is now similar to Western Europe. In Eastern Europe, however, mortality rates remain higher.

All-cause and AIDS mortality decreased across all regions from 2001 to 2020. Next to universal access and early start of ART, possible explanations include the improved management of major communicable co-infections (e.g., tuberculosis and hepatitis C), rapid/same day initiation of ART and increasing integration of assessment and health care for non-communicable diseases.^15^^,^^27^^,^^28^ In addition, overall, AIDS and non-AIDS mortality for the various (sub-) regions within the definition of Western Europe in this study (i.e., Northern Europe, Southern Europe and Western Europe) were very similar, in line with findings from the same cohort published previously for the years 2001–2011.^17^ However, both remained higher in Eastern Europe.^4^ Eastern Europe had the highest proportion of unknown or poor IVS compared to the other regions, which might partly be explained by a delayed implementation of guidelines recommending early ART as well as the higher proportion of late HIV diagnoses in this region.^29^^,^^30^ Although Eastern Europe is still behind, a significant progress has been made in regards of IVS status in 2016–2020 vs. in 2001–2010, which is reflected in the reduction in AIDS mortality. However, there is still an urgent need for measures to be taken to speed up the process for Eastern Europe to reach Western European or Central European 2020 levels without an unreasonable additional delay. ART-naïve status at baseline was associated with a higher mortality only in Eastern Europe, which suggests higher levels of late HIV diagnosis and later start of ART compared to the other two regions of Europe. To substantiate this hypothesis, we analyzed the median CD4 cell-count at ART-treatment initiation during follow-up in our cohort: those in Eastern Europe had a median CD4-cell count of 240 (IQR 154–354) at ART-initiation, compared to 316 in Central Eastern (IQR 210.50–456.50) and 317 (234.25–448.75) in Western Europe. In Western Europe, being ART-naïve appears to protect against death, which is likely due to those ART-naive less often having a history of injection drug use (14.2% vs. 24.6%), "ever HCV" (17.2% vs. 37.2%) or AIDS pre-baseline (5.4% vs. 29.6%) compared to those on ART. Conversely, baseline characteristics of people with HIV in Eastern Europe, where being ART-naïve is a risk factor for death, include almost half with a history of injecting drug use as risk factor for HIV acquisition. These people are more frequently co-infected with hepatitis C and likely represent a substantially disadvantaged socio-economic group.^31^^,^^32^ Interestingly, female gender appeared to be associated with lower all-cause and AIDS-related mortality, with its effect being larger in Central Eastern and Eastern Europe. These findings merit further detailed investigations but are beyond the scope of this manuscript.

The most important risk factors for all-cause mortality in the adjusted multivariable model were poor or unknown IVS, being off ART, and pre-baseline as well as incident active TB during follow-up. All of these have previously been described as major risk factors for mortality, in particular in Eastern Europe.^33^ The effect of active TB on mortality declined over time, as has been shown to be the case in the Swiss HIV Cohort study, where being on ART and HIV care had the most pronounced impact on risk of developing active TB.^34^

Stratified by region, unknown IVS status was only associated with a larger risk of mortality in Eastern Europe. This could be explained by unknown IVS status being a marker of sub-optimal access to laboratory facilities, primarily in earlier time periods before 2015 or even 2010, when the rate of measurement of CD4-cell count and HIV-RNA viral load was lower in Eastern Europe compared to Central Eastern and Western Europe (Supplementary Table S5).

The high number and the widespread geographical distribution of participating clinics is a major strength of the EuroSIDA cohort study. However, the collaborating clinics are in general major urban clinics and therefore not necessarily representative of any nation's entire HIV health care system, but rather reflects best standard. This suggests an inherent risk of selection bias and needs to be taken into account when interpreting the results of the present study. The proportion of unknown and missing causes of death within our cohort has been reduced by using previously developed techniques.^16^^,^^17^ In addition, the ample number of person-years of follow-up and events ensured robust analysis. A limitation of our study is that some important mortality predictors have so far not been systematically collected, including socioeconomic status, alcohol abuse, current substance abuse, while some key explanatory variables such as smoking had a substantial proportion of missing data. Another limitation is that differences in ART regimens by region and changes over time were not taken into account. Also, no information was available on TB drug resistance, availability of diagnostics and differences in management. Furthermore, loss to follow-up is increasing over time, possibly exacerbated during the recent years due to the COVID19-pandemic, and relatively higher in Eastern Europe, which might introduce bias leading to a potential misestimation of mortality differences.

### Conclusion

All-cause and AIDS mortality improved over time across regions but remained higher in Eastern Europe.

Patients immunologic-virologic status was generally better in Western and Central Eastern Europe over the entire follow-up period, whereas Eastern Europe had the highest proportion of people with poor or unknown status. These findings emphasize the need to improve HIV care with a focus on early HIV diagnosis, early ART, ART adherence and retention in care, especially in Eastern Europe. Special attention should be paid to prevention, early diagnosis and proper management of active TB as it is associated with significantly increased mortality.

## Contributors

CK, ET, SS, LP, JR and OK designed the study and analysis plan. ET did the statistical analyses under supervision of JR and with support for data interpretation by CK, LP, and OK. CK and OK coordinated the study. CK wrote the first draft of the manuscript. All authors contributed to data collection, interpreted data and critically reviewed and commented on the draft report. All authors have approved the final version of the report.

## Data sharing statement

The datasets (and related data dictionaries) used and/or analyzed during the current study are available from the corresponding author on reasonable request.

## Declaration of interests

OK, BS, AC, GM have research grants, personal fees for lectures and consultancy, meeting support from Gilead, MSD and ViiV outside the submitted work. AC has received consulting fees participation on data monitoring board, personal fees for lectures and consultancy, meeting support from Gilead, MSD Jannsen Cilag and ViiV outside the submitted work. CK received payment for a lecture from Gilead outside the submitted work. ET, SS, LØ, GF, SM, KK, AK, JT, JR, RZ, FM, SW, JG, DP, LP, and JR declare no competing interests.
