# Supplementary material for: All-cause and AIDS-related mortality among people with HIV across Europe from 2001 to 2020: impact of antiretroviral therapy, tuberculosis and regional differences in a multicentre cohort study
Source: Lancet Reg Health Eur. 2024 Jun 25;44:100989. doi: 10.1016/j.lanepe.2024.100989 (PMC11259909; doi:10.1016/j.lanepe.2024.100989)
Supplement: Supplementary Tables [file mmc1.docx]

# Supplementary material

Supplementary Table 1. Risk factors for all-cause mortality, stratified by time-period of follow-up

| **Risk factor** | **Level** | **aIRR(95% CI)** | **aIRR (95% CI)** | **aIRR* (95% CI)** |
| --- | --- | --- | --- | --- |
|  |  | **2001–2010** | **2011–2015** | **2016–2020** |
| Age | per 1 year older | 1·06 (1·05, 1·07) | 1·07 (1·06, 1·08) | 1·06 (1·05, 1·07) |
| Gender (ref: Male) | Female | 0·77 (0·66, 0·9) | 0·97 (0·79, 1·19) | 0·66 (0·54, 0·82) |
| HIV transmission risk (ref: MSM) | IDU | 2·2 (1·9, 2·56) | 2·15 (1·73, 2·68) | 1·8 (1·46, 2·22) |
|  | Heterosexual contact | 0·94 (0·79, 1·11) | 1·04 (0·82, 1·32) | 1·11 (0·88, 1·39) |
|  | Other/unknown | 1·12 (0·89, 1·42) | 0·91 (0·63, 1·32) | 1·11 (0·79, 1·56) |
| Region (ref: Western) | Central Eastern | 1·07 (0·89, 1·29) | 1·02 (0·78, 1·32) | 0·89 (0·69, 1·14) |
|  | Eastern | 1·51 (1·25, 1·82) | 1·58 (1·27, 1·98) | 1·38 (1·09, 1·74) |
| Immunologic/Virologic status (ref: Good (CD4≥500 VL<200)) | Poor (CD4≤350 VL>200) | 6·8 (5·63, 8·22) | 10·25 (7·68, 13·68) | 8·48 (6·32, 11·36) |
|  | Intermediate (between poor and good) | 2·58 (2·17, 3·06) | 3·81 (3·12, 4·65) | 2·54 (2·15, 3) |
|  | Unknown | 4·34 (3·27, 5·76) | 3·78 (2·26, 6·32) | 1·93 (0·86, 4·33) |
| Current ART history (ref: On ART) | Off ART | 2·59 (2·23, 3·01) | 1·83 (1·35, 2·47) | 2·44 (1·67, 3·56) |
|  | ART naïve | 1·12 (0·89, 1·4) | 1·15 (0·81, 1·65) | 1·48 (0·88, 2·48) |
| Active TB history (ref: No history of TB) | History of TB, pre-baseline | 1·61 (1·32, 1·97) | 1·21 (0·9, 1·64) | 1·41 (1·08, 1·84) |
|  | Incident TB during follow-up | 5·27 (4·01, 6·93) | 2·5 (1·74, 3·59) | 2·26 (1·53, 3·34) |
| Smoking history (ref: Never smoker) | Former smoker | 1·01 (0·84, 1·21) | 1·56 (1·18, 2·07) | 1·15 (0·88, 1·51) |
|  | Current smoker | 1·27 (1·07, 1·51) | 2·07 (1·59, 2·7) | 1·84 (1·42, 2·38) |
|  | Unknown | 0·79 (0·64, 0·98) | 0·63 (0·43, 0·92) | 0·94 (0·68, 1·3) |

* The multivariable model for AIDS-related mortality was adjusted for age, gender, mode of HIV transmission, region, IVS, current ART history, time-updated active TB history, smoking history and time-period·

IRR-Incidence Rate Ratio; aIRR–adjusted incidence Rate Ratio; IDU–Injecting Drug Use; MSM–Men having sex with men; ART-Antiretroviral therapy; AIDS–acquired immunodeficiency syndrome; TB-tuberculosis; VL-HIV-RNA viral load

Supplementary Table 2. Risk factors for all-cause mortality in multivariable adjusted time-updated Poisson regression models, stratified by region of care

| **Risk Factor** | | **All-cause mortality** | | |
| --- | --- | --- | --- | --- |
|  |  | **West** | **Central East** | **East** |
|  |  | **aIRR (95% CI)** | **aIRR (95% CI)** | **aIRR (95% CI)** |
| Age | per 1 year older | 1·07 (1·06, 1·07) | 1·06 (1·04, 1·07) | 1·04 (1·03, 1·05) |
| Time Period (ref: 2001 – 2010) | 2011 – 2015 | 0·8 (0·71, 0·91) | 0·72 (0·53, 0·96) | 0·81 (0·64, 1·02) |
|  | 2016 – 2020 | 0·77 (0·68, 0·88) | 0·66 (0·48, 0·91) | 0·82 (0·64, 1·06) |
| Gender (ref: Male) | Female | 0·85 (0·75, 0·97) | 0·56 (0·4, 0·79) | 0·68 (0·54, 0·86) |
| HIV transmission risk (ref: MSM) | IDU | 2·23 (1·98, 2·52) | 1·54 (1·12, 2·11) | 2·19 (1·38, 3·47) |
|  | Heterosexual contact | 0·93 (0·81, 1·07) | 1·03 (0·71, 1·48) | 1·63 (1·02, 2·61) |
|  | Other/unknown | 1·02 (0·85, 1·24) | 1·36 (0·86, 2·15) | 1·13 (0·54, 2·37) |
| Immunologic/Virologic status (ref: Good) | Poor | 7·5 (6·38, 8·81) | 8·5 (5·85, 12·35) | 7·44 (5·24, 10·55) |
|  | Intermediate | 2·96 (2·63, 3·32) | 2·26 (1·67, 3·05) | 2·54 (1·81, 3·57) |
|  | Unknown | 2·18 (1·3, 3·67) | 3·21 (1·37, 7·54) | 4·47 (3·03, 6·58) |
| Current ART history (ref: On ART) | Off ART | 2·71 (2·33, 3·16) | 2·15 (1·46, 3·16) | 2·02 (1·53, 2·67) |
|  | ART naïve | 0·52 (0·32, 0·86) | 0·73 (0·35, 1·55) | 1·33 (1·03, 1·71) |
| Active TB history (ref: No history of TB) | History of TB, pre-baseline | 1·34 (1·12, 1·6) | 1·99 (1·37, 2·89) | 1·76 (1·3, 2·39) |
|  | Incident TB during follow-up | 2·72 (1·87, 3·96) | 3·02 (1·77, 5·16) | 4·14 (3·16, 5·41) |
| Smoking history (ref: Never smoker) | Former smoker | 1·07 (0·92, 1·25) | 1·42 (0·94, 2·13) | 1·6 (1·08, 2·38) |
|  | Current smoker | 1·47 (1·27, 1·7) | 1·88 (1·33, 2·66) | 1·87 (1·29, 2·7) |
|  | Unknown | 0·79 (0·66, 0·95) | 0·75 (0·41, 1·38) | 0·85 (0·5, 1·47) |

The multivariable models was adjusted for age, gender, mode of HIV transmission, time-period under follow-up, and IVS, ART history, active TB history, and smoking history.

aIRR: adjusted incidence Rate Ratio; 95%CI: 95% confidence interval; IDU: Injecting Drug Use; MSM: Men having sex with men; ART: Antiretroviral therapy; AIDS: acquired immunodeficiency syndrome; TB: tuberculosis;

Supplementary Table 3. Person-years of follow-up per age group by region stratified by time period

|  |  | **2001 – 2010** | | | **2011 – 2015** | | | **2016 – 2020** | | |
| --- | --- | --- | --- | --- | --- | --- | --- | --- | --- | --- |
| **Region** |  | **West** | **Central East** | **East** | **West** | **Central East** | **East** | **West** | **Central East** | **East** |
| Age group (in years) | 0–30 | 2056·449 | 1757·8782 | 3555·32101 | 809·7029 | 698·5626 | 1788·5503 | 340·9966 | 396·4736 | 421·7194 |
|  | 31–40 | 17050·511 | 4020·2984 | 2645·85626 | 4152·2519 | 2846·5845 | 4626·8200 | 2725·5852 | 2259·8385 | 3905·4839 |
|  | 41–50 | 27332·906 | 2506·6448 | 1011·55921 | 14184·5010 | 2803·2416 | 1655·5565 | 9691·5455 | 3243·7509 | 2911·4497 |
|  | 51–60 | 12328·216 | 1096·9637 | 327·87406 | 12525·4702 | 1209·0185 | 596·0602 | 17831·1376 | 1929·8261 | 750·4093 |
|  | >60 | 5482·960 | 382·5079 | 75·37577 | 6103·3785 | 629·7906 | 146·2149 | 9240·6160 | 958·5270 | 212.084 |

Supplementary Table 4. Risk factors for all-cause and AIDS-related mortality in univariable and multivariable adjusted time-updated Poisson regression models, using 29^th^ February 2020 as cut-off

| **Risk Factor** | | **All-cause** | | **AIDS** | |
| --- | --- | --- | --- | --- | --- |
|  |  | **IRR (95%CI)** | **aIRR (95%CI)** | **IRR (95%CI)** | **aIRR (95%CI)** |
| Age | per 1 year older | 1·03 (1·02, 1·03) | 1·06 (1·06, 1·07) | 0·98 (0·97, 0·99) | 1·03 (1·02, 1·04) |
| Time Period (ref: 2001 – 2010) | 2011 – 2015 | 0·74 (0·68, 0·82) | 0·79 (0·72, 0·88) | 0·45 (0·36, 0·56) | 0·54 (0·43, 0·68) |
|  | 2016 – 2020 | 0·65 (0·59, 0·71) | 0·65 (0·58, 0·73) | 0·26 (0·2, 0·34) | 0·41 (0·3, 0·55) |
| Gender (ref: Male) | Female | 0·71 (0·64, 0·78) | 0·79 (0·71, 0·88) | 0·75 (0·61, 0·93) | 0·63 (0·5, 0·81) |
| HIV transmission risk (ref: MSM) | IDU | 2·16 (1·97, 2·37) | 2·06 (1·85, 2·3) | 2·27 (1·83, 2·82) | 1·17 (0·91, 1·51) |
|  | Heterosexual contact | 0·95 (0·85, 1·05) | 1·01 (0·9, 1·14) | 1·44 (1·14, 1·81) | 1·2 (0·93, 1·54) |
|  | Other/unknown | 1·2 (1·01, 1·42) | 1·08 (0·91, 1·28) | 1·18 (0·79, 1·77) | 0·9 (0·59, 1·38) |
| Region (ref: Western) | Central Eastern | 0·79 (0·7, 0·9) | 1·01 (0·88, 1·15) | 1·33 (1·02, 1·74) | 1·43 (1·08, 1·9) |
|  | Eastern | 1·52 (1·38, 1·69) | 1·46 (1·29, 1·66) | 3·76 (3·11, 4·55) | 2·2 (1·7, 2·85) |
| Immunologic/Virologic status (ref: Good) | Poor | 8·95 (7·97, 10·06) | 7·59 (6·64, 8·67) | 67·91 (45·61, 101·11) | 37·69 (24·78, 57·33) |
|  | Intermediate | 3·19 (2·88, 3·53) | 2·86 (2·57, 3·17) | 9·06 (6·05, 13·57) | 6·92 (4·6, 10·4) |
|  | Unknown | 4·61 (3·78, 5·6) | 4·32 (3·47, 5·36) | 45·76 (29·14, 71·85) | 23·06 (14·24, 37·36) |
| Current ART history (ref: On ART) | Off ART | 4·04 (3·61, 4·52) | 2·42 (2·14, 2·75) | 7·35 (5·93, 9·1) | 2·37 (1·88, 3) |
|  | ART naïve | 1·56 (1·33, 1·84) | 1·18 (0·98, 1·4) | 3·57 (2·72, 4·68) | 1·06 (0·78, 1·43) |
| Active TB history (ref: No history of TB) | History of TB, pre-baseline | 1·78 (1·54, 2·04) | 1·48 (1·28, 1·71) | 2·73 (2·06, 3·61) | 2·27 (1·7, 3·03) |
|  | Incident TB during follow-up | 5·61 (4·66, 6·74) | 3·43 (2·82, 4·17) | 22·94 (18·08, 29·1) | 11·4 (8·7, 14·93) |
| Smoking history (ref: Never smoker) | Former smoker | 1·31 (1·14, 1·49) | 1·16 (1·01, 1·33) | 1·01 (0·77, 1·33) | 1·02 (0·76, 1·35) |
|  | Current smoker | 1·82 (1·62, 2·05) | 1·58 (1·39, 1·79) | 1·33 (1·04, 1·69) | 1·07 (0·82, 1·39) |
|  | Unknown | 0·86 (0·73, 1·01) | 0·81 (0·69, 0·96) | 0·73 (0·52, 1·02) | 0·81 (0·58, 1·14) |

* Multivariable models were adjusted for age, gender, mode of HIV transmission, time-period under follow-up, region, and time-varying IVS, current ART, time-varying active TB, smoking status.

IRR: univariate, unadjusted incidence Rate Ratio; aIRR: adjusted incidence Rate Ratio; 95%CI: 95% confidence interval; IDU: Injecting Drug Use; MSM: Men having sex with men; ART: Antiretroviral therapy; AIDS: acquired immunodeficiency syndrome; TB: tuberculosis; VL: viral load

Supplementary Table 5. CD4 and HIV-Viral Load measurement rate, per region and time-period (measurements/year of follow-up)

| Region | Measure | **2001 – 2010** | **2011 – 2015** | **2016 – 2020** |
| --- | --- | --- | --- | --- |
| West | CD4-cell count | 3·28 (3·27, 3·3) | 2·24 (2·23, 2·26) | 1·54 (1·53, 1·55) |
| East | CD4-cell count | 1·69 (1·66, 1·72) | 1·56 (1·53, 1·58) | 1·25 (1·23, 1·28) |
| Central East | CD4-cell count | 2·46 (2·42, 2·49) | 2·09 (2·06, 2·13) | 1·43 (1·41, 1·46) |
| West | HIV-RNA viral load | 3·23 (3·22, 3·24) | 2·4 (2·38, 2·42) | 1·89 (1·88, 1·91) |
| East | HIV-RNA viral load | 0·97 (0·95, 1) | 1·25 (1·23, 1·28) | 1·27 (1·25, 1·3) |
| Central East | HIV-RNA viral load | 1·79 (1·77, 1·82) | 1·71 (1·68, 1·74) | 1·33 (1·3, 1.35) |
